# Transcriptome and metabolite analysis identifies nitrogen utilization genes in tea plant (*Camellia sinensis*)

**DOI:** 10.1038/s41598-017-01949-0

**Published:** 2017-05-10

**Authors:** Wei Li, Fen Xiang, Micai Zhong, Lingyun Zhou, Hongyan Liu, Saijun Li, Xuewen Wang

**Affiliations:** 1 National Center for Tea Improvement-Hunan Branch, Tea Research Institute, Hunan Academy of Agricultural Science, 702 Yuanda Road, Changsha, 410125 P. R. China; 2 Germplasm Bank of Wild Species, Kunming Institute of Botany, Chinese Academy of Sciences, 132 Lanhei Road, Kunming, 650201 P. R. China; 3 Hunan Provincial Key Laboratory of Phytohormones and Growth Development, Hunan Agricultural University, Changsha, 410128 P. R. China; 4 Department of genetics, 120 E Green Street, Athens, GA 30602 United States

## Abstract

Applied nitrogen (N) fertilizer significantly increases the leaf yield. However, most N is not utilized by the plant, negatively impacting the environment. To date, little is known regarding N utilization genes and mechanisms in the leaf production. To understand this, we investigated transcriptomes using RNA-seq and amino acid levels with N treatment in tea (*Camellia sinensis*), the most popular beverage crop. We identified 196 and 29 common differentially expressed genes in roots and leaves, respectively, in response to ammonium in two tea varieties. Among those genes, *AMT*, *NRT* and *AQP* for N uptake and *GOGAT* and *GS* for N assimilation were the key genes, validated by RT-qPCR, which expressed in a network manner with tissue specificity. Importantly, only *AQP* and three novel DEGs associated with stress, manganese binding, and gibberellin-regulated transcription factor were common in N responses across all tissues and varieties. A hypothesized gene regulatory network for N was proposed. A strong statistical correlation between key genes’ expression and amino acid content was revealed. The key genes and regulatory network improve our understanding of the molecular mechanism of N usage and offer gene targets for plant improvement.

## Introduction

Nitrogen (N) is an essential element in biological systems, including DNA, proteins, and amino acids. N utilization efficiency (NUE) is the key issue in N usage in plants though the definition of NUE may differ. In agriculture, NUE can be simply calculated as the ratio of gained biomass or produce with supplied N to that without supplied N. However, NUE in plants is very low^1^. More than half of active N is lost^2^, which results in considerable threats to our environment via water pollution and biodiversity changes. The excessive N in the environment costs at least 100 million dollars per year in the European Union^3^. Therefore, increasing global attention to reduce N emission has risen, and a 20% improvement of NUE by 2020 was proposed^3^. One of the best solutions to this problem is to make full use of or to engineer NUE genes^3, 4^.

The inorganic ammonium and nitrate are the two major sources of mineral N in soil. To date, the N uptake in some plants is known to be regulated by the ammonium transporter gene *AMT*, the nitrate transporter gene *NRT*, and the aquaporin protein gene *AQP* ^5–8^. Multiple *AMT* and *NRT* homologous genes may exist in some plant species^5, 9^. In *Arabidopsis*, eleven homologous *NRT* genes were identified but they play very different roles including nitrate inducible, repressible or constitutive^9^. *AMT* expression is dependent on N signal in roots and spatial arrangement^10^ while *NRT* expression is regulated by the N signal of the whole plant in *Arabidopsis* ^11^. A reciprocal regulation between homologs of *AMT* or *NRT* and between *AMT* and *NRT* was reported^9, 11, 12^. Nitrate can enhance the uptake of ammonium while the latter will repress the uptake of nitrate in rice^13, 14^. However, the reverse is true in tea root^15^. AQP has been found to involve in water transportation by upregulating its gene expression in supplied N source in poplar^16^, rice^17^ and barley^7^. N uptake is also associated with phytohormones such as cytokinins, abscisic acid and auxin^18^, or transcription factors *osDOF18* in rice^19^.

Tea is the most popular non-alcoholic natural beverage in the world. *Camellia sinensis* (L.) O. Kuntzes is widely grown in more than 52 countries as an important commercial crop for leaf production, from which tea is made. China and India produce the most tea, with 1.9 million and 1.2 million metric tons of tea in 2013, respectively, according to statistics at http://www.statista.com. Nitrogen fertilizer is critical for increasing the tea leaf production in farming, which is also true for other crops^20^. N fertilizer is widely applied in tea farming. However, less than one quarter of applied N can be used by the wooden tea crop, meaning that the remaining will potentially be returned to the environment^21^. Therefore, the improvement of NUE in tea plant may be the key to these problems.

To date, few studies were published on kinetics or physiology of N uptake in tea. Most tea varieties prefer ammonium than nitrate at the N uptake rate^15^, the kinetics of N absorption, content of free amino acid, biomass and the related enzymes in the N assimilation^22–24^. A recent study showed that in tea roots, nitrate inhibits ammonium uptake while ammonium increases the uptake of nitrate in the early stage^15^. However, little is known regarding NUE genes in tea.

Amino acids are important storage compounds of N in plants^4^. After the uptake of active form of N, the glutamine (Gln) synthetase gene (*GOGAT*) and glutamic acid (Glu) synthetase gene (*GS*) play roles in N assimilation into amino acids in plants^5^. Higher glutamine concentration in roots represses the *AMT* and *NRT* expression in *Arabidopsis* ^14, 25^. In tea, Glu and theanine are the major amino acids. The high amount of theanine, a tea specific amino acid, is usually used as an index of good tea quality. Theanine is mainly synthesized in the roots^26^, and is then transferred to the aerial part of the plant. In general, theanine accounts for approximately 1–2% of the dry weight of tea leaves^27^, and accounts for approximately 60–70% of the total amount of free amino acids in tea^26^. However, the gene encoding theanine synthetase is still unclear by now. We discovered a tea variety Baojinghuangjin tea 1# (HJ) with higher levels of amino acids and a higher leaf yield than the common used variety Fudingdabaicha (FD) after applying N fertilizer^28^, suggesting that the variety HJ has a higher NUE. Therefore, HJ will be an ideal tea variety to be investigated for gene candidates responsible for NUE compared to other teas.

RNA-seq technology has been widely used in biological studies to sensitively discover transcripts on a single-nucleotide level and to quantify gene expression levels. For example, RNA-seq is a powerful approach for discovering key gene candidates in *Populus* ^29^, *Tabacum* ^30^, *Arabidopsis thaliana* and wheat^31, 32^. In *C*. *sinensis*, RNA-seq analysis has revealed some known genes in the secondary metabolic pathways, such as theanine biosynthesis^33^, and stresses, such as cold^34^, shade^35^ and drought^36, 37^. However, the key genes that control NUE in tea plant have not been reported. Because the whole tea plant genome sequence is not currently publicly available, we used RNA-seq to *de novo* identify NUE genes in this study.

In this study, we focused on NUE, especially on the genes responsible for N uptake from soil by roots and N assimilation into amino acids in tea plants. We used Illumina RNA-seq technology and amino acid measurement to identify genes and their regulation network associated with N transport and assimilation in response to ammonium fertilizer in the tea crop. Transcriptome analysis was conducted on both the leaves and roots with or without N treatment from two tea varieties, HJ and FD. In total, more than 100 million paired-end RNA-seq reads were generated, and approximately 20,000 unigenes were obtained by *de novo* assembly. The tea plant genes *AMT*, *AQP*, *GS* and *GOGAT* were identified as differentially expressed in the regulation of N uptake and cross talk in a network manner, with tissue specificity. Most importantly, we identified three novel genes differentially regulating N use in all tissues across tea varieties with predicted functions in stress, manganese binding and gibberellin (GA) regulated transcription factors. Combining with gene regulation and amino acid content, we found a strong correlation in statistics between gene expression and amino acid content. The shared and specific genes on NUE in tea plant can be applied to NUE improvement in tea and other plants.

## Results

### *De novo* assembly of *C*. *sinensis* transcripts

**Figure 1:**
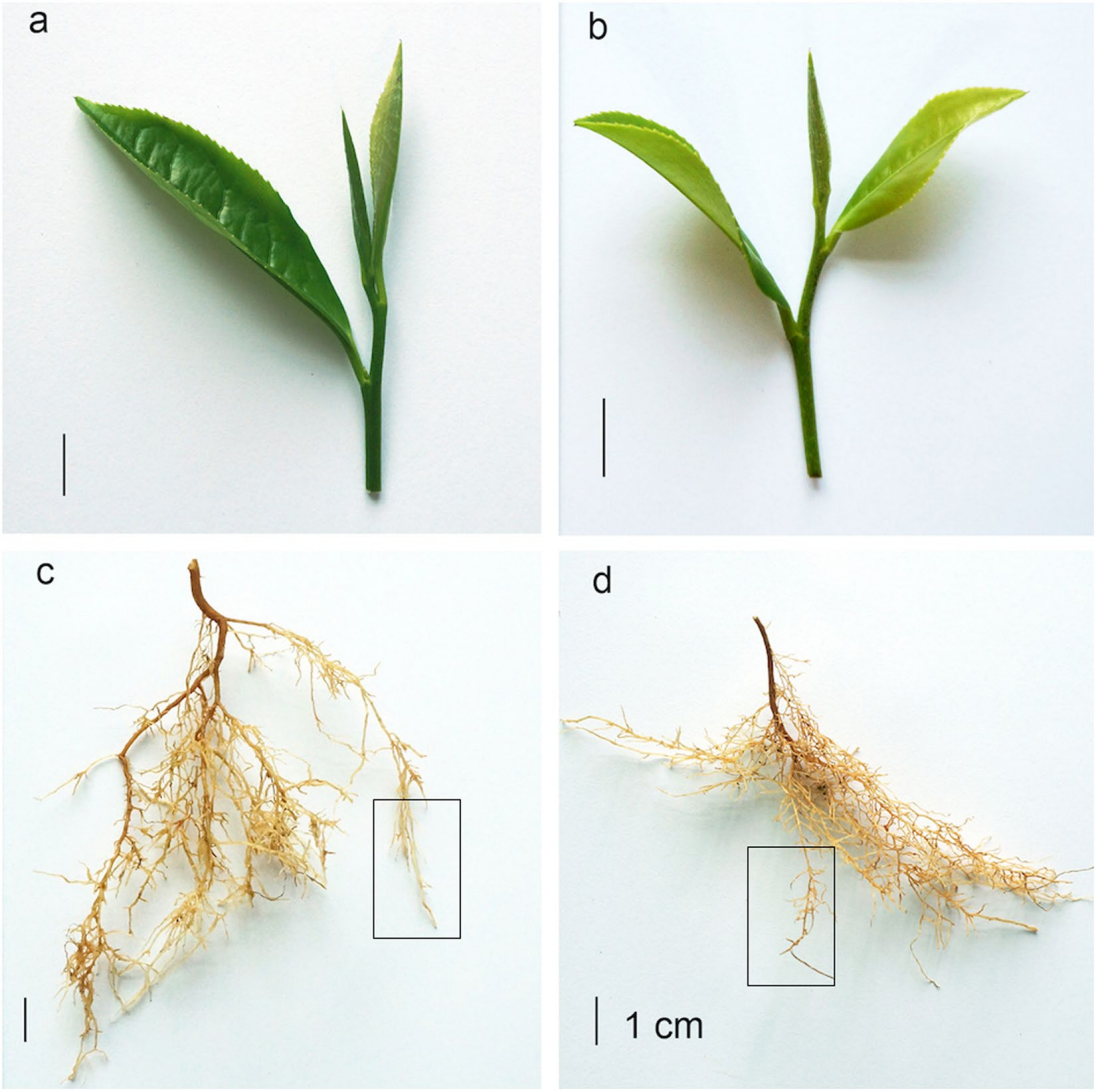
*C*. *sinensis* tea leaf and root samples. Images show the used tea samples without ammonium treatment. (**a**,**b**) are the representative leaves of the *C*. *sinensis* varieties HJ and FD. Only the buds and leaves were used in the study. (**c**,**d**) are the representative roots of the *C*. *sinensis* varieties HJ and FD. The boxed roots represent the types of roots used for analysis.

We treated two *C*. *sinensis* varieties, HJ and FD, with ammonium and extracted mRNA from young leaf tissues and tender roots with and without ammonium treatment (Fig. 1). The transcriptomes of these samples were then examined using RNA-seq technology. A total of 100,232,165 and 102,115,437 clean reads were generated from the tissues of HJ and FD, respectively. Then, the reads were *de novo* assembled into transcripts by using Trinity software (version 201308)^38^. We obtained very similar total numbers of unigenes: 194,519 and 198,118, in HJ and FD, respectively. The average lengths of the unigenes were 528 and 510 bp in HJ and FD, respectively (Table S1). The RNA-seq reads and the assembly are publicly available at NCBI under the master accession number SRP077092.

### Gene functional annotation

The unigenes were annotated by similarity searching (E-value ≤ 10^−10^ for Pfam, E-value ≤ 10^−5^ for others) against the following databases: NR, Swiss-Prot, GO, COG, KOG, and KEGG. A total of 86,856 (44.65%) and 82,305 (41.54%) unigenes were annotated in the HJ and FD, respectively (Table S2).

Based on sequence homology, 60,544 (31.12%) unigenes of HJ were distributed among three main GO categories: cellular component (16 functional groups, dominated by ‘cell part’ and ‘cell’), molecular function (17 functional groups, dominated by ‘catalytic activity’ and ‘binding’), and biological process (20 functional groups, dominated by ‘metabolic process’ and ‘cellular process’) (Fig. S1). For FD, 52,026 (26.26%) unigenes were also classified by GO, and the subgroup numbers and dominant terms of each category were the same as those in HJ. In both varieties, more unigenes were classified in biological process than in either of the other two classes.

To further analyze the functions of unigenes in *C*. *sinensis*, 19,389 and 18,157 unigenes in HJ and FD were annotated by the KEGG Automatic Annotation Server, respectively. These unigenes were enriched in 118 KEGG pathways. In total, 6,765 (34.89%) and 6,329 (34.86%) unigenes were found in metabolism pathways in HJ and FD, respectively. In HJ, 1,639 (8.45%) unigenes were enriched in the pathways of amino acid metabolism compared to 1,627 (8.96%) unigenes in FD (Fig. S2).

### Differentially expressed genes in response to ammonium

The differentially expressed genes (DEGs) were measured according to the RNA-seq reads’ abundance and were normalized to fragments per kilobase length per million reads (FPKM). After ammonium treatment, 1,997/635 DEGs were up/down-regulated in the roots while 3,596/306 DEGs were up/down-regulated in the leaves of HJ. Meanwhile, 4,195/345 DEGs were up/down-regulated in the roots while 220/223 DEGs were up/down-regulated in the leaves of FD (Table 1). More DEGs were found in the leaves than in the roots of HJ, while the reverse was true in FD (Table 1). The difference of DEGs may account for the different genetically inherited regulation, including N use efficiency, in the FD and HJ tea varieties.

**Table 1:** The regulation of differentially expressed genes after ammonium treatment.

	Roots			Leaves		
	up	down	total	up	down	total
*C*. *sinensis* variety HJ	1,997	635	2,632	3,596	306	3,902
*C*. *sinensis* variety FD	4,195	345	4,540	220	223	443

The data show the numbers of DEGs after ammonium treatment compared to the blank control. The definition of DEGs was set as more than a two-fold change in FPKM in the RNA-seq data. The phrase up, down, and total represent up, down, and total regulated expression, respectively.

**Figure 2:**
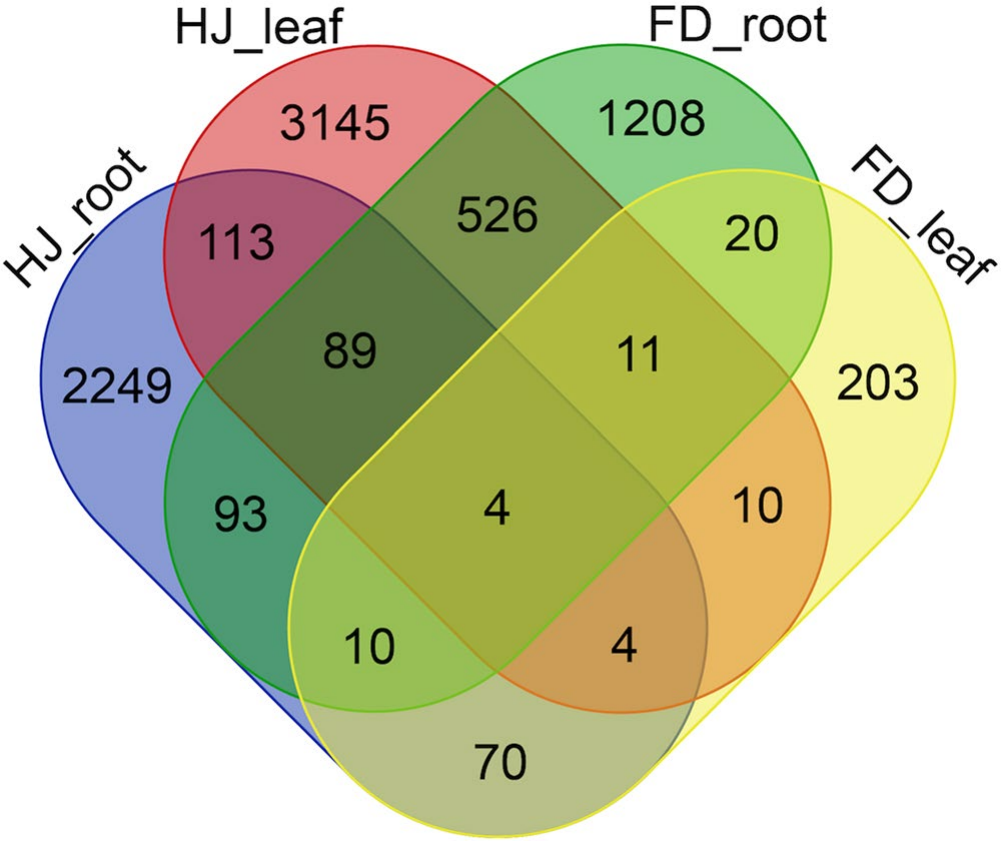
The differentially expressed genes in the roots and leaves of the *Camellia* HJ and FD in response to ammonium. The number of DEGs between the leaves and roots of two *C*. *sinensis* varieties in response to ammonium treatment are shown in this diagram. The phrases HJ_root, HJ_leaf, FD_root and FD_leaf represent the roots or leaves of the HJ variety and the roots or leaves of the FD variety, respectively.

To compare expression differences in response to ammonium treatment in both varieties, we BLASTed DEGs in one variety against the unigene assembly of the other variety reciprocally to find the homologous genes and then assessed whether the genes were DEGs in the other variety. After comparing the DEGs in the same tissues, we found 196 common DEGs in the roots and 29 common DEGs in the leaves of both varieties (Fig. 2).

To understand the network pathways involved by the 196 common DEGs in roots, we mapped these DEGs against the known pathways of *Arabidopsis* and poplar in KEGG database, respectively. Results showed the DEGs were mapped into more pathways of poplar (43 pathways) than *Arabidopsis* (36 pathways), indicating ammonium responses in tea plant was more similar to poplar tree than *Arabidopsis* (Table S4). The pathways were distributed rather scattered, suggesting ammonium treatment affected the functions of multiple pathways. The top three DEGs-enriched pathways were the pathway of biosynthesis of secondary metabolites, metabolism of terpenoids and polyketides, and biosynthesis of cofactor and vitamins (Fig. S3). The most affected pathway was biosynthesis of secondary metabolites (Fig. S3). To overview of these pathways, we highlighted the node, where DEGs mapped, in the pathway in the black color and provided the link to the pathway gene in poplar and *Arabidopsis* (Table S4).

**Figure 3:**
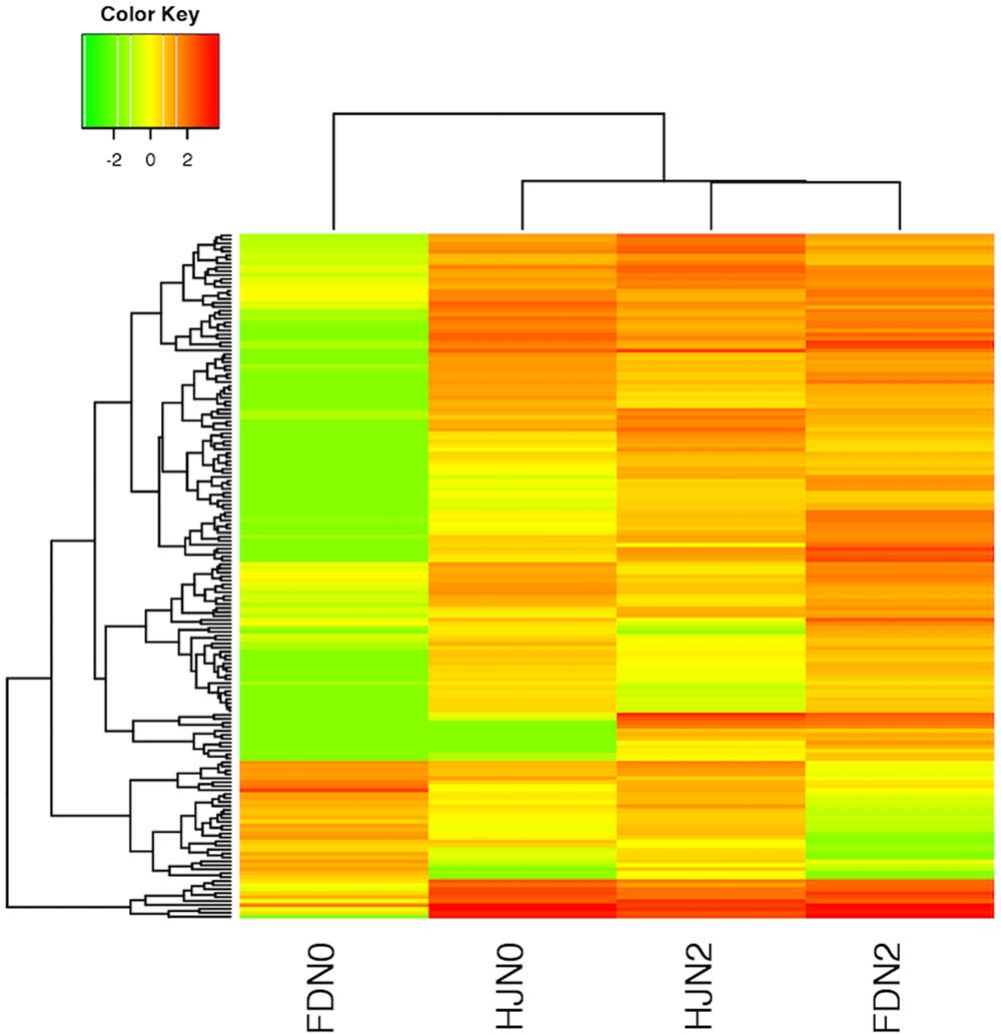
The expression patterns of shared differentially expressed genes in the roots of *Camellia*. The heatmap represents the relative expression levels of 196 common DEGs in the roots of the *Camellia* varieties HJ and FD. Expression levels were determined in FPKM using RNA-seq and were log2-transformed. The tree diagram represents the similarity between the four samples or between the common genes. “HJN0” and “FDN0” represent *C*. *sinensis* HJ/FD treated with 0 g of (NH_4_)_2_SO_4_ (the control), and “HJN2” “FDN2” represent *C*. *sinensis* HJ/FD treated with 22 g (NH_4_)_2_SO_4_.

Four shared DEGs were identified in both the leaves and roots across both varieties after treatment (Fig. 2), of which the predicted functions were annotated as “stress response (stress),” “manganese binding ion (Mn), nutrition storage,” “transcription factor (TF), a gibberellin regulated protein” and “aquaporin protein encoded by *AQP*” (Table S3). We further research for any homologs of these four genes and did not find any homolog in the sequence data of unigenes and transcript spliced isoforms. This finding suggested that the four key shared genes were responsible for common ammonium responses. To our knowledge, this study describes the first time that this set of four genes was discovered in response to ammonium in tea plant. The cluster of DEG expression patterns in roots revealed a high similarity between the HJ variety before ammonium control and FD after ammonium treatment (Fig. 3), indicating that the HJ variety had a higher N response, meaning a higher NUE in HJ than FD.

Of the common DEGs, 14 unigenes were identified as N response candidates, as these genes were annotated as ammonium-, or glutamine- or nitrate-related genes. The nitrogen response related genes (N gene) included *AMT*, *NRT*, *AQP* and *GS* (Table 2), which are known to be involved in ammonium transport and assimilation into amino acids in other plants^4^.

**Table 2:** Differentially expressed genes shared in two *Camellia* varieties.

Tissue	Gene ID	Annotated function
Root	c124953.graph_c1	ammonium transporter (AMT)
	c112070.graph_c0	nitrate transporter (NRT)
	c95905.graph_c0	aquaporin protein (AQP)
	c94357.graph_c0	glutamine synthetase (GS)
Leaf	c95905.graph_c0	aquaporin protein (AQP)
	c123483.graph_c 0	nitrate transporter (NRT)

DEGs were involved in the process of the transport and assimilation of ammonium in both *Camellia* varieties. The gene ID is the name of the unigene in the assembly of the *Camellia* variety HJ.

### The expression levels of N genes in response to ammonium

**Figure 4:**
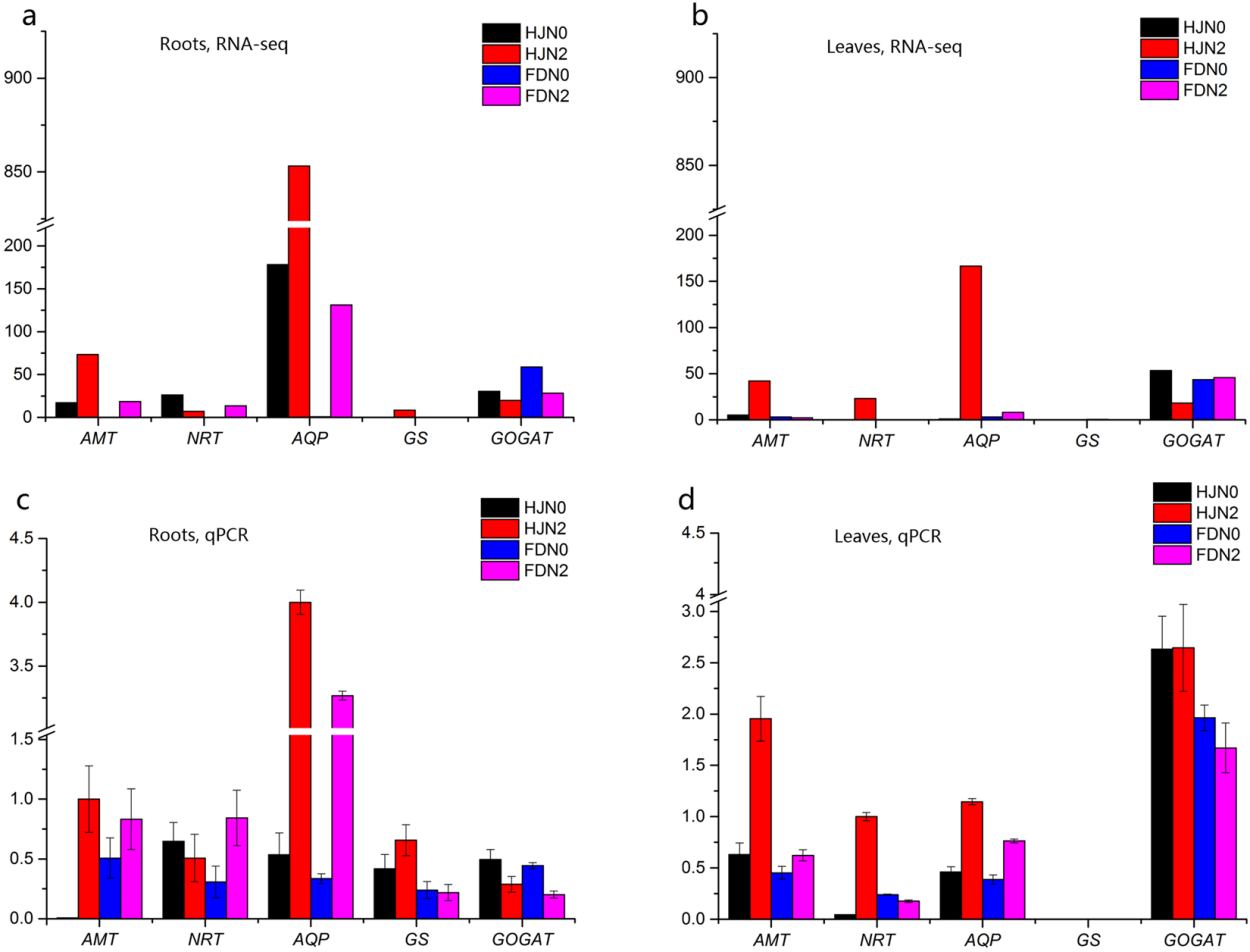
The expression levels of nitrogen associated in *Camellia* in response to ammonium. (**a**) The expression levels of genes in FPKM in RNA-seq data in the roots. (**b**) The expression levels of genes in FPKM in RNA-seq data in the leaves. (**c**) The relative expression levels of genes in the roots confirmed by RT-qPCR. (**d**) The expression levels of genes in the leaves confirmed by RT-qPCR. “HJN0” “FDN0” represent *C*. *sinensis* HJ/FD treated with 0 g of (NH_4_)_2_SO_4_ (the control). “HJN2” “FDN2” represent *C*. *sinensis* HJ/FD treated with 22 g of (NH_4_)_2_SO_4._ *AMT*, *NRT*, *AQP*, *GS* and *GOGAT*, representing the genes of the ammonium transporter, nitrate transporter, aquaporin protein, glutamine synthetase, and glutamic acid synthetase, respectively.

We further analyzed the expression levels in FPKM of N-related genes identified between the two varieties of *C*. *sinensis* (Fig. 4). Different regulation patterns of N genes were found in the two varieties. In the control, the expression levels of *AMT*, *NRT* and *GS* was very low while that of *AQP* was high (Fig. 4a,b). Ammonium induced an increase in *AMT*, *GS* and *AQP* expression levels in both tissues of both varieties (Fig. 4a,b). *NRT* expression displayed distinct patterns in different tissues of the two varieties. *NRT* was very lowly expressed in the leaves in the controls of both varieties. After ammonium treatment, *NRT* expression was significantly induced in the leaves of HJ. In the roots, *NRT* expression was decreased in HJ but induced in FD. Ammonium also down-regulated the Glu synthetase gene *GOGAT* expression except in the leaves of FD (Fig. 4a,b), indicating an inhibition of NH_4_^+^ by product from its catalysis Gln to Glu. To validate the RNA-seq results, we used the RT-qPCR to examine these key gene expression levels in the roots and leaves of HJ and FD (Fig. 4c,d). In HJ and FD, the trend of expressions of the five genes were similar to the results from RNA-seq data in leaves and roots of tea plant.

### Regulation network of amino acid content in response to ammonium input

Comparing the N-associated metabolites with the N genes is interesting. We measured the amounts of total amino acid, theanine and Glu in the tea leaves (Table 3). Before ammonium treatment, similar amounts of total amino acids were detected in both varieties, but higher amounts of theanine (P < *0*.*05*) and Glu (P < *0*.*01*) were present in the HJ variety compared to FD (Table 3). After treatment, HJ contained significantly higher (P < *0*.*001*) amounts of total amino acids, Glu and theanine compare to FD (Table 3). The major amino acid in HJ tea leaves was theanine, accounting for nearly 70% of the amino acids before treatment and approximately 60% after treatment (Table 3). The relatively decreased percentage of theanine after treatment indicated a higher additive increase of the other amino acids.

**Table 3:** The amino acid content in *Camellia* leaves before and after ammonium treatment.

	*C*. *sinensis* variety HJ		*C*. *sinensis* variety FD	
	N0 (mg·g^−1^)	N2 (mg·g^−1^)	N0 (mg·g^−1^)	N2 (mg·g^−1^)
Glutamic acid (Glu) (percentage)	4.54 ± 0.12** (10.07%)	6.20 ± 0.10*** (9.21%)	3.94 ± 0.15 (8.89%)	2.99 ± 0.09 (5.21%)
Theanine (percentage)	31.74 ± 0.55* (70.39%)	40.12 ± 0.54*** (59.59%)	29.84 ± 0.71 (67.36%)	35.11 ± 0.38 (61.23%)
Total amino acids	45.09 ± 0.63	67.33 ± 0.46***	44.3 ± 0.50	57.34 ± 0.29

The above table shows the content of amino acids in the tea leaves of HJ and FD. N0 represents the control of *C*. *sinensis* HJ/FD, and N2 represents the *C*. *sinensis* HJ/FD treated with (NH_4_)_2_SO_4_. The percentage is the amount of amino acids relative to total amino acids. *, **, and *** represent statistical t-test *P values* less than significance levels 0.05, 0.01, and 0.001, respectively, in the HJ variety compared to the FD variety at the same N condition.

**Figure 5:**
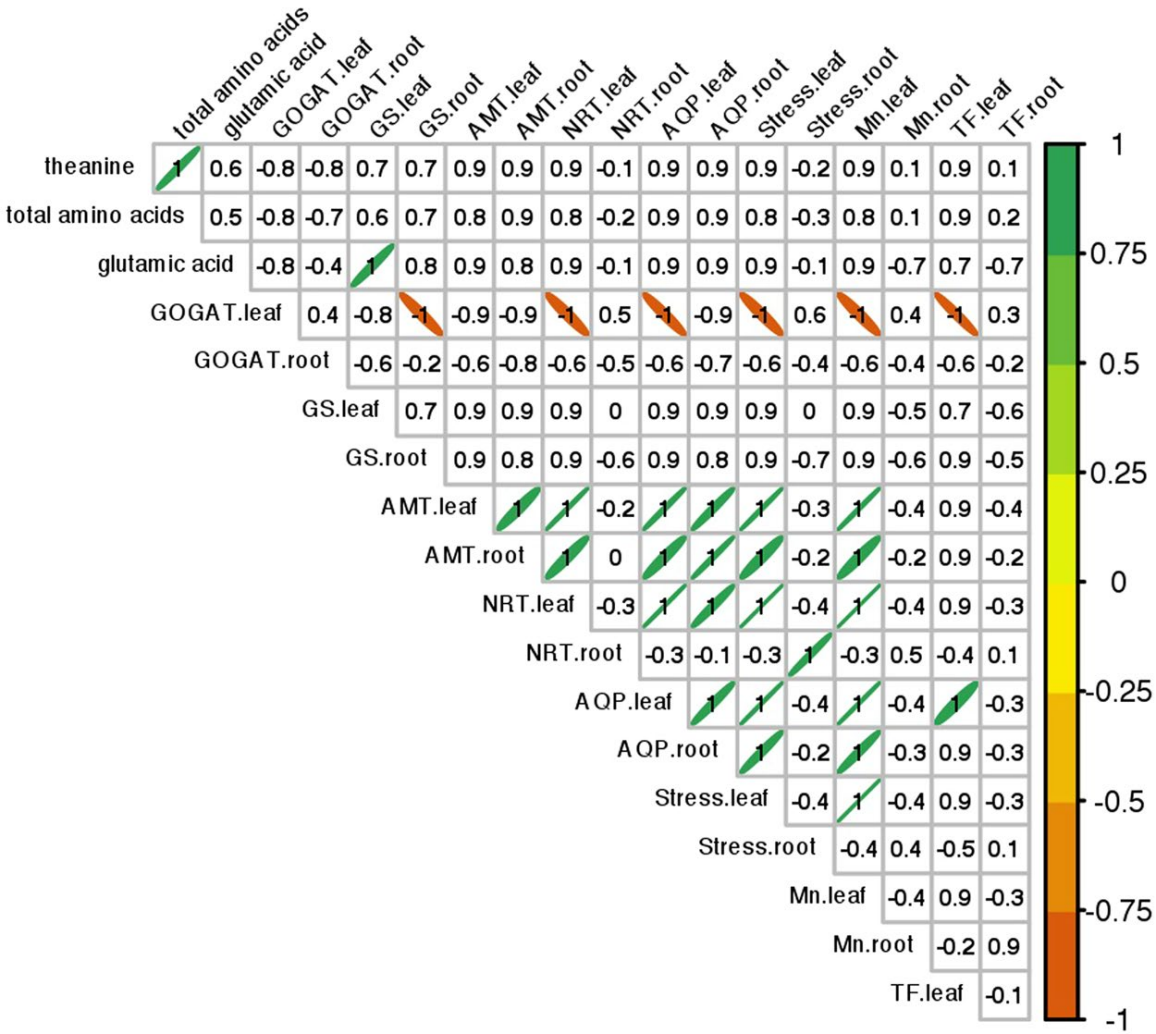
Correlations between amino acid content and gene expression in the *Camellia* leaves. This diagram shows the correlations between amino acid contents in leaves and gene expression levels. The gene name followed by “.leaf” or “.root” included i) four shared DEGs associated with stress, manganese (Mn), transcript factors (TF) and *AQP* in both the roots and leaves of two Camellia varieties, ii) differentially expressed N uptake genes *AMT* and *NRT*, and iii) Glu metabolism pathway genes *GS* and *GOGAT*. The number in each cell represents the coefficient. The significant (P < *0*.*05*) correlations were marked with an ellipse, and the direction denotes a positive or negative correlation.

To understand the DE gene expression and their impact on amino acid content, we analyzed the correlation between amino acids and gene expression levels using Pearson correlation statistics. An obvious NUE regulatory network was identified (Fig. 5). The content of theanine positively correlated to the total amount of amino acids, and *GS* expression positively correlated to Glu in leaves (coefficient = 1, P < *0*.*05*), with the highest coefficient of 1 suggesting the strongest correlation effect. Gene *AMT* expression positively co-expressed with *NRT*, *AQP*, and the remaining three common DEGs in both leaves and roots (Fig. 5). *NRT* genes in the leaves co-expressed with *AQP*, the stress gene and the Mn gene identified in this study, while *NRT* only correlated with the stress gene in roots. *AQP* positively correlated with the common genes associated with stress and Mn in leaves and roots while also co-expressed with TF in leaves. *GOGAT* expression in leaves was negatively correlated to *NRT*, *AQP* and three common DEG expression levels in leaves (P < *0*.*05*) (Fig. 5). *GOGAT* expression in leaves was also negatively correlated to *GS* expression in roots, which may be a remote regulation caused by Gln transferring between the roots and leaves.

## Discussion

Due to the concern regarding the impact of active N runoff from our environment^3^, the NUE is a hot topic. The NUE includes N uptake and assimilation into plants, and the current key step is to discover NUE genes^4^ and their potential variants. Although tea is the most popular beverage, little has been known about NUE genes until recently. Luckily, several genes for N usage in plants have been reported independently in model plants, such as *Arabidopsis* and rice, and were reviewed by ref. 4. The genes *AMT*, *AQP* and *NRT* play roles in N uptake in roots^5–8^, and the *GS* gene is important in N assimilation in leaves^5^. In this study, we used RNA-seq to investigate the N genes based on expression changes in response to ammonium (NH_4_^+^), a typical preferred active N, in the wooden crop tea. We identified more than 2000 DEGs in response to NH_4_^+^ in two different tissues, leaves and roots, of tea plants. These DEGs, including previously reported genes, provided candidate gene resources for further investigating ammonium transport and assimilation in tea and other plants.

Currently, several RNA-seq-based reports on *C*. *sinensis* mainly focus on the metabolisms i.e. theanine metabolism^33, 39, 40^, and stress response such as cold^34, 41^. Those studies contributed some gene sequences in tea plant and identified some preliminary candidate genes. In contrast to those studies, we designed the N studies before and after ammonium treatment in the roots and leaves of two tea varieties: a recently identified variety HJ with a higher N response and a widely-grown variety FD with an average N response. After several years of investigation, we have confirmed that the HJ variety has a higher free amino acid content in the spring tea leaves compared to other tea plant after applying ammonium. The higher amino acid content indicates a higher quality of tea in the industry. The interesting phenotype encouraged us to investigate the N genes in HJ with the aim for higher NUE gene variants. After comparing the genes’ responses in the roots and leaves of each variety, we identified shared DEGs: 196 in the roots plus 29 in the leaves, with a variety of specific DEGs in response to ammonium during tea leaf production (Fig. 2). This finding suggested that ammonium induced more gene responses in roots than in leaves in the tea varieties.

The DEG expression patterns explained the different NUE and growth stages of the tea varieties HJ and FD. There were more DEGs in the leaves of HJ compared to its roots while there were fewer DEGs in the FD leaves than in the FD roots in response to NH_4_^+^ (Table 1). This difference may be due to genetic variance or different leaf starting stages in the early spring. Previous research showed that HJ was an early sprouting tea variety. The stage of one spread leaf and a bud was 5–7 days earlier than FD^28^. Therefore, our treatment was set to two weeks so that both varieties produced one bud and two leaves after the treatment to reduce the difference caused by leaf developmental stages. However, the greater expression of DEGs in the leaves of HJ compared to FD may still partially result from the earlier leaf development in HJ compared to FD (Table 1).

**Figure 6:**
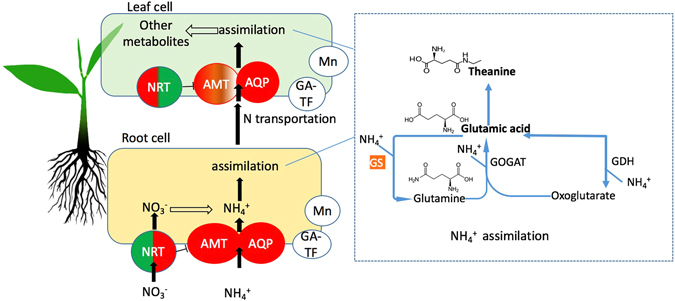
Nitrogen uptake, assimilation in roots and leaves of tea plant. Black and blue arrows represent the movement of ammonium and metabolite pathway, respectively. *AMT*, *NRT*, *AQP*, *GS* and *GOGAT*, represent the genes of the ammonium transporter, nitrate transporter, aquaporin protein, glutamine synthetase, and glutamic acid synthetase, respectively. Mn and GA-TF represent new identified manganese binding protein and gibberellin related transcript factors, respectively. Colors shading the protein show the up-regulated (red) or down-regulated (green) expression. Presence of both red and green colors shows varied regulation between the tea variety HJ and FD.

We identified the tea homologs of N genes reported in other plants. To illustrate the gene regulation, we proposed a regulation network (Fig. 6) by adding our new finding to previous understanding^4^. AMT is an ammonium transporter in plants^5, 6, 42, 43^. In this study, after ammonium treatment, the *AMT* gene was differentially up-regulated in the roots of both tea varieties. This result suggested a common *AMT* induction by NH_4_^+^ in the roots of tea plants. However, in the leaves, *AMT* was up-regulated (8-folds) in HJ while it was unchanged in FD. This difference in *AMT* level may result from genetic variance of *AMT* or its regulation, which accounts for the higher NUE in HJ.

The *NRT* gene, known for specific nitrate^44^ and auxin^45^ uptake, was down-regulated in the roots and up-regulated in the leaves of HJ after ammonium treatment. In contrast, in FD, *NRT* was up-regulated in the roots of FD while it was unchanged in leaves. It was reported that ammonia increases the uptake of nitrate in tea roots^15^, but it lasts for a very short time. Our results are from two weeks adaptation to ammonium instead of less than hour in previous publication^15^. We proposed that this result could be caused by the reciprocal repression of *NRT* and *AMT* ^12, 46^. The higher *AMT* expression in HJ indicated a stronger repression of *NRT*, leading to decreased *NRT* expression in the HJ roots (Table 3, Fig. 4). Alternatively, a higher N uptake suggests a higher conversion to other forms of N, i.e. NO_3_^−^ through metabolism, which requires increased NO_3_^−^ transportation, meaning also increasing *NRT* expression. We speculated that the bio-directional regulations dynamically controlled *NRT* expression, which explained the net increase of *NRT* in FD roots and HJ leaves because relatively low *AMT* had less of a repressive effect on *NRT* (Table 3, Fig. 4). The difference of *NRT* expression in roots and leaves was also affected by long-distance transport of N. It is possible that *NRT* expression may also be affected by allelic differences and/or different gene regulation efficiencies between the HJ and FD varieties.

*AQP* encodes a water channel protein for water and ammonia (NH_3_) transportation^4, 47^. We found that *AQP* expression was significantly up-regulated (5- to 20- fold) in all tissues after ammonium treatment (Fig. 4). Accumulated NH_4_^+^ in plant cells will lead to ammonium toxicity^1^. The observed up-regulation of *AQP* in both the leaves and roots in both tea varieties may function to buffer the toxicity of NH_4_^+^ by water uptake to maintain an osmotic balance. The AQP channel is an alternative way for plants to take up N in the NH_3_ format^48, 49^, which is supported by a recently revealed structural function of the AQP protein^47^ and was demonstrated as in barley roots^7^. *AQP* expression in HJ was higher (greater than 8-fold) compared with that of FD in both tissues in the control and the treatment groups, indicating a higher N uptake capability through *AQP* in HJ. This finding also accounts for the observed higher NUE in HJ compared to FD. Previous studies showed that some mineral nutrients such as P and Ca were up-taken by AQP^8^. Interestingly, we found the co-occurrences of both increased expression of ion Mn binding gene and increased N up taking, which will be further analyzed in the subsequent investigation.

In this study, we identified four common DEGs in both the leaves and roots across tea varieties in response to ammonium. One gene is *AQP*, discussed above. The remaining three genes are not well characterized. However, from the conserved sequences of the predicted amino acids of these genes, we predicted their functions as “stress response,” “manganese binding nutrition storage” and “transcription factor, a gibberellin regulated protein.” It is rational for genes to act in “stress response” and “nutrition storage” because NH_4_^+^ acts as a type of toxic stress and is also an essential element of nutrition. NH_4_^+^ induces hormone-related responses^1^, so one identified gene functioning as “transcription factor, a gibberellin regulated protein” will also be interesting for NUE. Few transcription factors, i.e., ZmDof1 in maize^50^, in N responses have been previously identified, and we have found a new one herein. Further studies will focus on the function of these common novel genes. Of course, some specific DEGs which expression change were exclusively in either FD or HJ variety may be potential candidates to interpret the difference in response to ammonium. In addition, the difference of root phenotype may contribute the difference in response to ammonium though we did not found extremely different change by eyes. We did identify several genes, which were related to root morphology, from the DEGs list such as the c119942.graph_c3 (Germin-like protein in roots, auxin, up-regulated), c126564.graph_c2 (transcription factor, a gibberellin regulated protein, selected in this article), c108112.graph_c0 (root cap, up-regulated).

The analysis of amino acid content revealed a higher N assimilation into amino acids in the leaves of HJ variety compared to FD after N-fertilizer input (Table 3). This was consistent with the significantly upregulated expression patterns of N uptake genes *AMT*, *AQP* and *NRT* in leaves, suggesting that these genes play import roles in N use efficiency. Previously study showed that the NH_4_^+^ uptaken is then assimilated into Gln by glutamine synthetase gene *GS* through Gln-Glu metabolizing cycles (Fig. 6) in roots, and then transported to leaves^51, 52^. The *GS* expression can be induced by the increased ammonium^53^ and that is consistent in our study. Our finding revealed that a strong positive correlation of GS gene expression with Glu level was in leaves. This suggested that *GS* expression in leaves plays major role in controlling dynamic level of Glu in leaves. We found that *GOGAT* expression was negatively correlated with the expression of *NRT*, *AQP* and three common DEG in leaves with ammonium supply. This may be a feedback repression of high NH_4_^+^ amount on the expression of *GOGAT* which also functions as to release NH_4_^+^ from Gln. Taken together, low expression of *GOGAT* and the higher expression of *AMT*, *AQP*, *NRT* and *GS* work together to promoting the NH_4_^+^ uptake and assimilation into amino acids. The higher expression levels of *AMT* and *GS* genes in HJ compared to FD (Fig. 4) suggested a higher capability of ammonium uptake and higher N assimilation in HJ than FD. The Glu content in the HJ variety increased while FD displayed a downward trend after N fertilizer treatment (Table 3). This result could be explained as a balance between Glu generation and its use as a substrate in other metabolism pathways, such as the theanine synthesis pathway^53^. Therefore, theanine and Glu content are regulated by both N transporter genes and additional genes, such as *GS* and *GOGAT*.

## Conclusion

Our study revealed that many DEGs control N usage efficiency in the tea plant *Camellia sinensis* for active N resources in the soil. The two *C*. *sinensis* varieties with different NUEs result from different and four shared genes’ expression in the roots and leaves. The *AMT*, *AQP*, *NRT*, *GOGAT* and *GS* genes are the key shared DEGs acting together to regulate ammonium uptake and assimilation. Some of the genes are directly correlated with N use in tea leaves. Future research on the role of the identified three novel common DEG N-responsive genes will be the first priority in elucidating the mechanism of high N use efficiency in plants. The study improved the understanding of the molecular regulations of NUE, and provided direct gene references for engineering and guiding the breeding selection of high nitrogen-efficient varieties of *C*. *sinensis*.

## Materials and Methods

### Materials

Cuttings from two *Camellia* tea varieties, HJ and FD, were propagated for new tea plants and grown to tea industrial size over 3 years. All of the plants were grown in the same experimental field at GPS coordinates 113°4′30.168″E and 28°12′20.580″N, and under the same conditions at the Hunan Tea Research Institute, in Changsha, China. HJ is much higher tea plant than FD. As the leaves and bud are considered, tea plant HJ has a much faster growing speed than FD.

### Ammonium treatment

Four propagated plants of each variety were treated with 22 g of (NH_4_)_2_SO_4_ (Sinopharm Chemical Reagent co., LTD, cat# 10002918, Shanghai, China) per 14.5 kg of dry soil, a commonly used range during farming, and four plants were used as experimental controls without (NH_4_)_2_SO_4_ treatment^22^. The available nitrogen amount in this soil used is 94.7 mg per kg dry weight. The treatment was conducted at the stage when the bud was 1–2 mm long and no new spread leaf was present at the top of a branch, two weeks after this point (in April 2014), the bud would spread into a new leaf. Leaves and roots were collected separately on the 15^th^ day after treatment. Leaf samples included the bud, the 1^st^ leaf and the 2^nd^ leaf positioned from the top to bottom, and young absorbing roots such as the latest and the second latest lateral roots were sampled (Fig. 1). Tissues were snap-frozen in liquid nitrogen and stored at −80 °C until processing. Two repeated experiments were performed. The relative water content in the growing soil was 50–70%.

### RNA extraction and sequencing

Samples from the two repeats were pooled and ground into powder in a mortar using liquid nitrogen. Total RNA was extracted from 80–120 mg of powder using a TIANGEN RNAprep Pure kit (TIANGEN, catalog # DP441) with our optimized procedures (pending patent CN104694531 A). The quality and quantity of total RNA were characterized on a 1% agarose gel and examined with a NanoDrop 2000c spectrophotometer (NanoDrop Technologies, Wilmington, DE, USA). The RNA integrity number (RIN) was assessed using an Agilent 2100 Bioanalyzer (Santa Clara, CA, USA). If the RIN was greater than 8.0, the RNA was used for subsequent Illumina library preparation.

mRNA was enriched from 15 μg of total RNA using an NEBNext poly (A) mRNA Magnetic Isolation Module (NEB, cat# E7490L) and AMPure® XP Beads (Beckman Coulter, Inc., cat# A63881). mRNA was cleaved into short fragments in buffer and was then indexed. The sequencing library was prepared using an NEBNext mRNA Library Prep Master Mix Set for Illumina (NEB, cat# E6110L) and NEBNext Multiplex Oligos for Illumina (NEB, cat# E7500). The library was then subjected to qPCR-based quantification using a Quantification Kit-Illumina GA Universal (Kapa, cat# KK4824). Paired-end 125-bp sequencing was performed for the qualified library on a HiSeq 2500 machine.

### Real-time quantitative PCR (RT-qPCR) assay

1–2 μg total RNAs were used for reverse transcription by FastQuant RT kit (TIANGEN, catalog # KR106). 1 μL RT product was used as template in the subsequent step. The cDNA was diluted to 200 ng/μL was used for the qPCR with SuperReal PreMix Plus (TIANGEN, catalog # FP205) on the Bio-Rad CFX96 realtime system. Three technical replicates were applied for the relative gene expression analysis. Reactions were performed at 95 °C for 15 min, 40 cycles of 95 °C for 10 s, and 60 °C for 32 s. β-actin was used as reference for qPCR data analysis. All primers for RT-qPCR are listed in Table S5.

### Transcript assembly, annotation and expression analysis

Raw reads generated from the Illumina HiSeq 2500 platform were preprocessed to a clean adaptor sequence. Reads with >5% unknown bases were filtered out, and low-quality reads (>20% of the bases with a quality score of 10) were removed. The remaining reads, called clean reads, from the same variety were combined and *de novo* assembled using Trinity software (version 201308)^54^ to construct transcripts. Clean reads were mapped back to check the transcript quality, and poorly supported transcripts were filtered out as previously described^54^. Unigenes were defined as the longest sequence in the assembly cluster called a component in Trinity^54^. Unigenes were then searched using BLAST with an E-value threshold of E-5 against the NR (NCBI non-redundant protein sequences), Swiss-Prot, GO (Gene Ontology), COG (Clusters of Orthologous Groups), KOG (euKaryotic Orthologous Groups), and KEGG (Kyoto Encyclopedia of Genes and Genomes) databases, and against the Pfam database by HMMER with an E-value of 1E-10. The RNA-seq reads and the assembly are publicly available at NCBI under the master accession number SRP077092.

### Amino acid detection and measurement

The leaf samples were fixed with boiling water steam for 90 sec, dried at 60 °C and then ground into a fine powder. In total, 5.0 g of powder was boiled in 100 ml of water for 15 min to extract amino acids, and the supernatant was then filtered through absorbent cotton. Two additional runs of extractions from the residue of the same powder were performed in 100 ml and 50 ml water. All of the extracted filtrate was merged and cooled to room temperature. The merged filtrate was then filtered through a 0.45-μm membrane, and the filtered liquid was collected for free amino acid detection using an amino acid analyzer (Hitachi, L-8800, Japan) according to the method described in China National Stand GB/T 5009.124-2003.

## Electronic supplementary material

Supplementary Information

Additional file, Table S4
