# Supplementary material for: Transcriptome and metabolite analysis identifies nitrogen utilization genes in tea plant (Camellia sinensis)
Source: Sci Rep. 2017 May 10;7:1693. doi: 10.1038/s41598-017-01949-0 (PMC5431998; doi:10.1038/s41598-017-01949-0)
Supplement: Supplementary file 1 — Supplementary Information [file 41598_2017_1949_MOESM1_ESM.pdf]

Transcriptome and metabolite analysis identifies nitrogen utilization genes in tea plant (*Camellia sinensis*)

Wei Li[1, 2, 3†], Fen Xiang[1†], Micai Zhong[2], Lingyun Zhou[1], Hongyan Liu[1], Saijun Li[1]*, and Xuewen Wang[2, 4]*

List of supplementary Information

Figure S1. The gene ontology classification of unigenes in the *C. sinensis* variety HJ

Figure S2. KEGG classification of unigenes in the *C. sinensis* variety HJ

Figure S3. The top three enriched KEGG pathway of differentially expressed common genes in roots

Table S1. Summary of RNA-seq reads and assembly in two *C. sinensis* varieties

Table S2. Annotation summary of unigenes in *C. sinensis*

Table S3. Shared differentially expressed genes in both the leaves and roots of the *C. sinensis* varieties in response to ammonium input

Table S4. The involved KEGG pathways of common DEGs in *Camellia* roots

Table S5. Primers for RT-qPCR

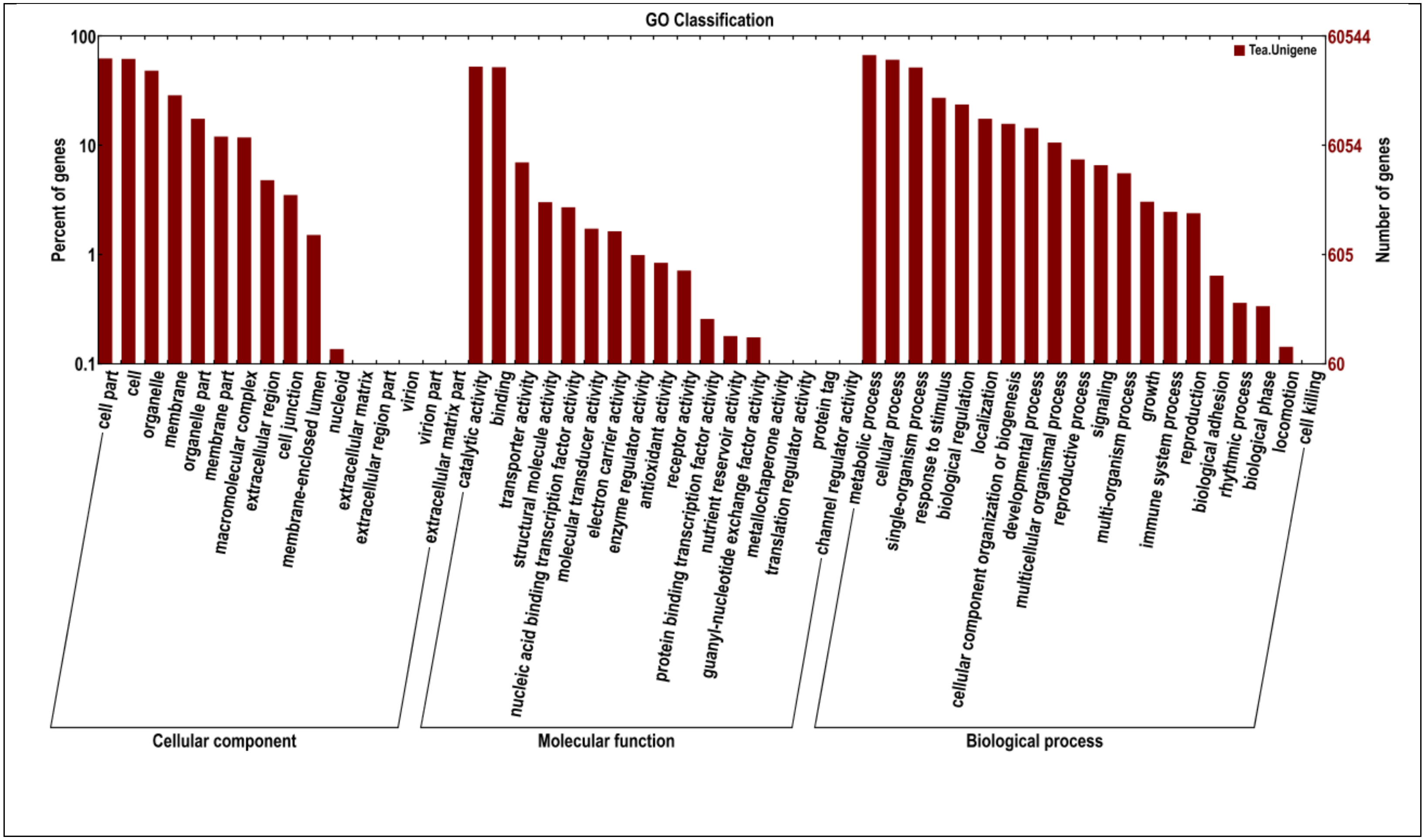

Figure S1. Gene ontology classification of unigenes in *C. sinensis* HJ

Abscissa represents the three major GO classes. The sub-classification was listed under the major categories. The ordinate represents the unigenes numbers in the right axis and the percentage of the total unigenes in the left axis.

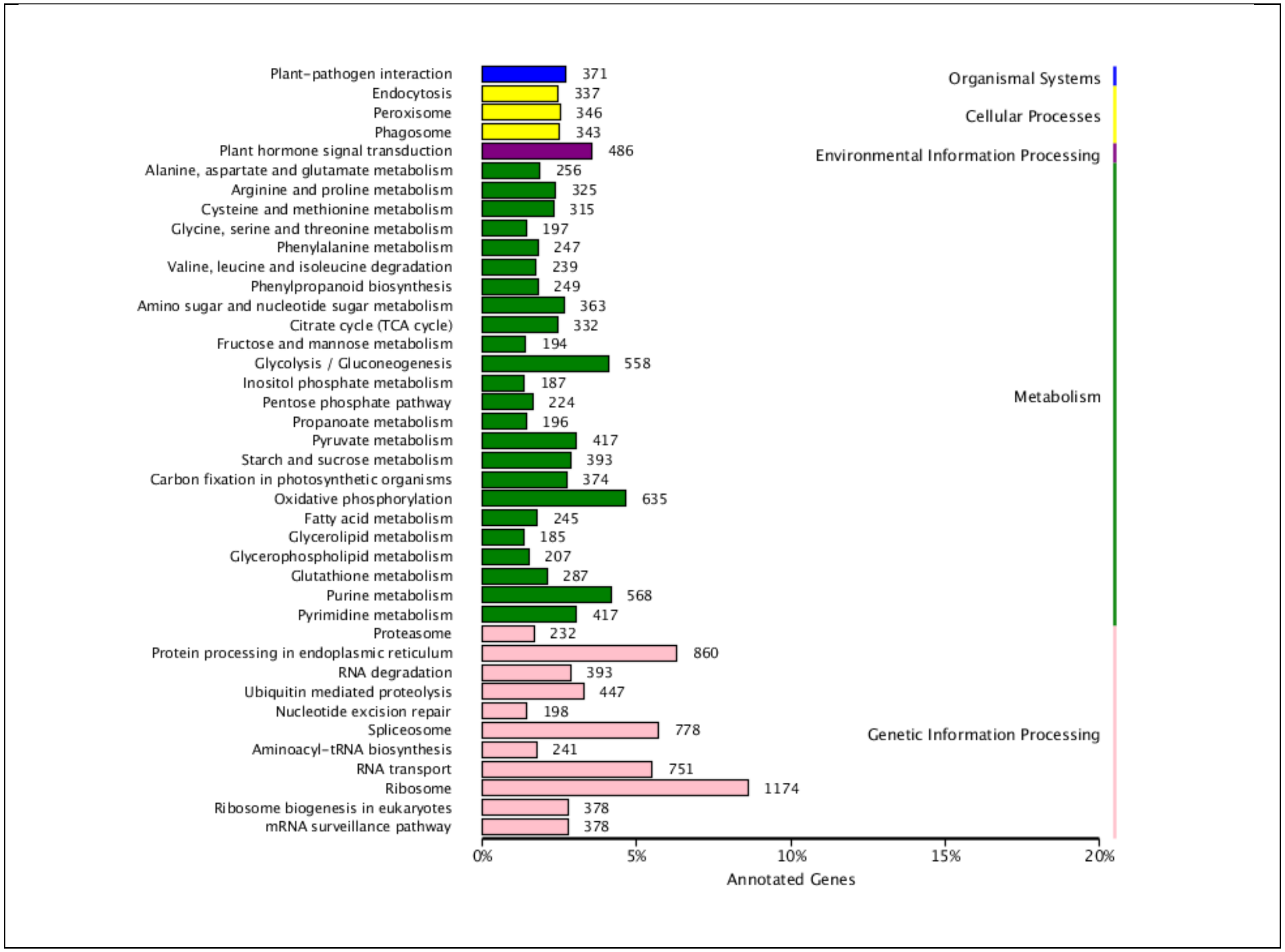

Figure S2. KEGG classification of unigenes in *C. sinensis* variety HJ

The five different colors represent the five categories of pathways. Each category is subdivided into several different classes. The vertical labels stand for the name of each pathway. The horizontal labels stand for the percentage of annotated pathway genes in the total number of unigenes annotated, and the number in the right of each bar is the number of unigenes in each pathway. The chart was generated for the top 40 pathways of the 118 total pathways.

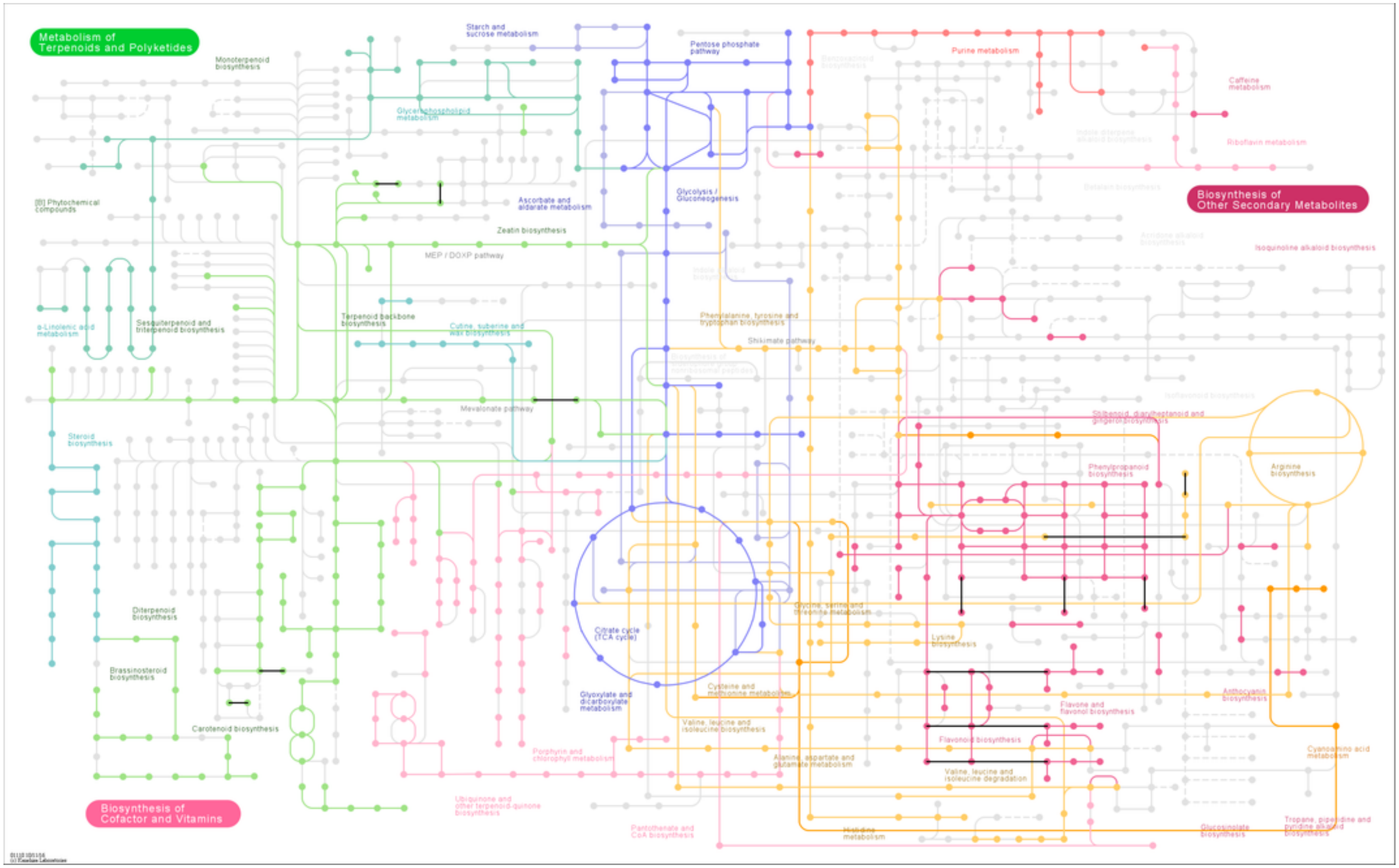

Figure S3. **The top three enriched KEGG pathway of differentially expressed common genes in roots**

Table S1. Summary of RNA-seq reads and assembly in two *C. sinensis* varieties

	HJ	FD
Total number of clean reads	100,232,165	102,115,437
Number of transcripts	331,260	331,355
Number of unigenes	194,519	198,118
Total length of unigene (bp)	102,628,478	101,125,646
Unigene N50 length (bp)	681	659
Average length of unigene (bp)	528	510

Data shows the statistical information of RNA-seq reads from Illumina Hiseq 2500 sequencing and assembly from software Trinity. The total number of clean reads is the sum of reads from both leaves and roots. HJ and FD represent *C. sinensis* varieties of Baojinghuangjin 1# and Fudingdabaicha, respectively.

Table S2. Annotation summary of unigenes in *C. sinensis*

databases	HJ		FD	
	Number	Percentage	Number	Percentage
COG	25,159	12.93	25,940	13.09
GO	60,544	31.12	52,026	26.26
KEGG	19,389	9.97	18,157	9.16
KOG	48,168	24.76	44,101	22.26
Pfam	47,749	24.55	45,891	23.16
Swiss-Prot	50,244	25.83	44,128	22.27
NR	84,614	43.50	78,291	39.52
all databases	86,856	44.65	82,305	41.54

The above table shows the numbers and percentage of unigenes annotated in databases. The number shown in the last row was the total count of the annotated genes after removing duplicated uingenes.

Table S3. Shared differentially expressed genes in both the leaves and roots of *C. sinensis* varieties in response to ammonium input

Gene ID	Predicted function
c128107.graph_c0	stress response
c119942.graph_c3	binding Manganese ion, nutrition storage
c126564.graph_c2	transcription factor, a gibberellin regulated protein
c95905.graph_c0	aquaporin protein

Table S4. The involved KEGG pathways of common DEGs in Camellia roots
(available in separate excel file)

Table S5. Primers for RT-qPCR

	Gene ID	Primer name	Primer sequence (5'--3'）
	Reference gene	β-actin1-F	GCCATCTTTGATTGGAATGG
		β-actin1-R	GGTGCCACAACCTTGATCTT
HJ	c124953.graph_c1	AMT.HJ.1F	GAGCCAAACACCACCTTGTT
		AMT.HJ.1R	AGGCGGAGAAGAGGAGGTAG
	c112070.graph_c0	NRT.HJ.1F	TTGGCCAACGTACATGAAAA
		NRT.HJ.1R	GATCCGGTTGTGAGCAGAAT
	c95905.graph_c0	AQP.HJ.1F	TGTTGGCACCCACTATGAAA
		AQP.HJ.1R	TCCGGTGTGACAGTATGGAA
	c94357.graph_c0	GS.HJ.2F	AGATCATTGAGGCCATCGAG
		GS.HJ.2R	ACGACGTATGGGTCCATGTT
	c114054.graph_c0	GOGAT.HJ.F	CAACATGGGCTTCGATCAGG
		GOGAT.HJ.R	TTGCTGCTGGTATGACTGGA
FD	c115533.graph_c0	AMT.FD.F	CAAAACCCAAGCTGACACGA
		AMT.FD.R	AGCCGCACTTACTACCTTGT
	c137324.graph_c0	NRT.FD.F	TTGGAGGAAAAGAGGGGCAT
		NRT.FD.R	GGGAAATGGTGCCGGAAAAT
	c68483.graph_c0(Same as HJ)	AQP.FD.1F	TGTTGGCACCCACTATGAAA
		AQP.FD.1R	TCCGGTGTGACAGTATGGAA
	c104005.graph_c0	GS.FD.F	CCCGACTGTTGGCATTTCTG
		GS.FD.R	CCTCATCGACTCGGTACTGT
	c133242.graph_c0	GOGAT.FD.F	CAACATGGGCTTCGATCAGG
		GOGAT.FD.R	TTGCTGCTGGTATGACTGGA
